# Central-Radial Artery Pressure Gradient after Cardiopulmonary Bypass Is Associated with Cardiac Function and May Affect Therapeutic Direction

**DOI:** 10.1371/journal.pone.0068890

**Published:** 2013-07-22

**Authors:** Jie Sun, Zhengnian Ding, Yanning Qian, Yong G. Peng

**Affiliations:** 1 Department of Anesthesiology, the First Affiliated Hospital with Nanjing Medical University/Jiangsu Province Hospital, Nanjing, China; 2 Department of Anesthesiology and Surgery, Shands Hospital at the University of Florida, Gainesville, Florida, United States of America; University of KwaZulu-Natal, South Africa

## Abstract

**Objective:**

To investigate the risk factors involved in radial-femoral artery pressure gradient after cardiac surgery.

**Methods:**

In this retrospective study, we reviewed 412 cardiac surgeries with both femoral artery pressure and radial artery pressure monitoring before cardiopulmonary bypass. 138 patients had radial-femoral artery pressure gradient after cardiopulmonary bypass (group P) but 263 were not (group N). Their hemodynamic data and other demographic data were analyzed.

**Results:**

Phenylephrine usage was 1.7±1.1 mg in group N and 2.9±1.2 mg in group P (P<0.001). Total adrenaline usage was 229.2±116.9 µg in group N and 400.6±145.1 µg in group P (P<0.001). SBP gradient was -4±3, 14±9, 10±4, 0±11 mmHg in group P and -3±3, 0±1, -1±9, -6±4 mmHg in group N after induction, during discontinuation of CPB, at the end of surgery and 1 postoperative day respectively. DBP gradient was 3±3, -1±9, 4±5, 0±8 mmHg in group P and 3±3, 5±2, 7±5, 0±8 mmHg in group N after induction, during discontinuation of CPB, at the end of surgery and 1 postoperative day respectively. MAP gradient was 1±2, 4±6, 6±4, 0±8 mmHg in group P and 1±2, 3±1, 1±4, -2±5 mmHg in group N after induction, during discontinuation of CPB, at the end of surgery and 1 postoperative day respectively. Significant arterial pressure gradient emerged during discontinuation of CPB and at the end of surgery, which was more obvious in group P(P<0.01). CI was 2.0±0.3, 2.3±0.4,2.3±0.4, 2.2±0.4 L/min/m^2^ in group P and 2.1±0.3, 2.8±0.5,2.8±0.5, 2.8±0.5 L/min/m^2^ in group N at baseline, after discontinuation of CPB, at the end of surgery and the first postoperative day (P<0.001).

**Conclusion:**

Detecting the exact central artery pressure is most important when patients have artery pressure gradients after cardiac surgery. Use inotropic agents to improve cardiac output, avoiding excessive vasoconstriction might reduce artery pressure gradient.

## Introduction

Direct intra-radial arterial pressure monitoring is often used in cardiac surgery because of the possible intensity of the hemodynamic changes as well as the need to assess the detailed hemodynamic parameters during the operation. However, a central-radial artery pressure gradient may happen after cardiopulmonary bypass (CPB) surgery, which in some patients may last for a significant long time after CPB. Systemic vascular resistance will be underestimated and therapeutic strategy is possible to be misleading in condition of significant artery pressure gradient [1]. To know an exact central artery pressure is very important for the anesthesiologists and the surgeons to evaluate the cardiac function, vital organ perfusion, calculate the detailed hemodynamic data and administrate corresponding vaso-active agents.

The exact mechanism of central-radial artery pressure gradient is still to be determined. Some prospective studies reported that there was no relationship between the magnitude of the pressure gradient and type of cardioprotection, bypass duration, temperature and systemic vascular resistance [2,3]. However, other researchers indicated that marked arteries constriction due to increased sympathetic nervous system contributed to the damped transmission of the pressure pulse to radial artery and therefore intensify radial-central pressure gradient [4]. In addition, decreased artery wall elasticity, deep hypothermia, and radial artery diameter were also confirmed to be causes of pressure gradient [5–7]. Despite the above possible etiologies, the mechanism of artery pressure gradient may be controversial and multifactorial. In this study, we reviewed 412 patients who received both radial artery pressure and femoral artery pressure monitoring at the very beginning, and 138 patients had radial-femoral artery pressure gradient after cardiopulmonary bypass. We tried to find out the risk factors involved in the artery pressure gradient postoperatively.

## Materials and Methods

Our research protocol was approved by the institutional review board and institutional review board waived the need for written informed consent from the participants (Human Study Protection Board, Nanjing Medical University). The cardiac anesthesia case records from Jan 1^st^, 2002 to Dec 31^st^ 2012 were screened and those patients with both direct femoral and radial artery pressure monitoring were collected in our research.

**Figure 1:**
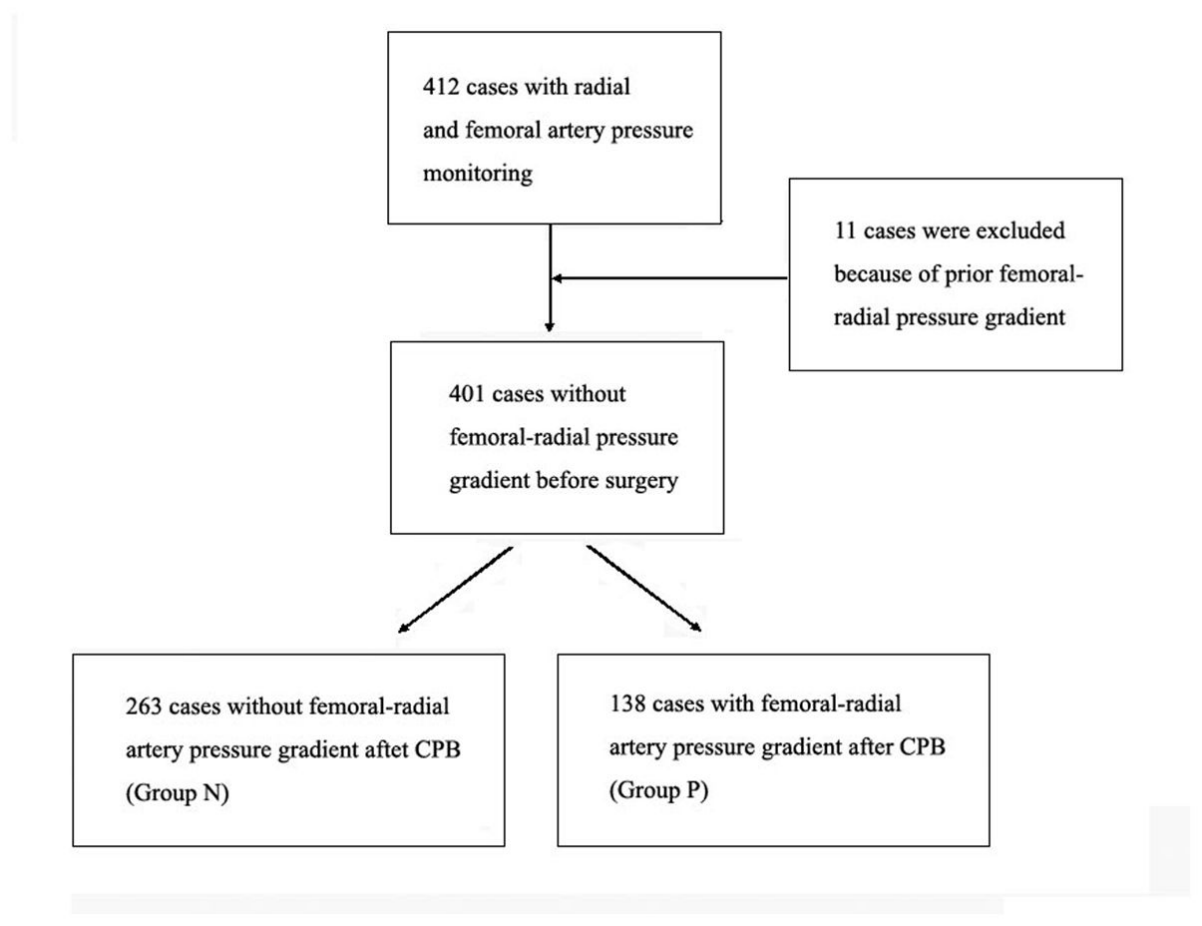
In this chart box, the first box suggested that 412 patients received both femoral artery pressure and radial artery pressure monitoring before cardiopulmonary bypass.

In this retrospective study, 412 patients received both femoral artery pressure and radial artery pressure monitoring before cardiopulmonary bypass. Of which, 11 patients were excluded because of a prior radial-femoral artery pressure gradient and the rest of patients did not have radial-femoral artery pressure gradient before the surgery. 263 patients did not have radial-femoral artery pressure gradient after CPB (Group N). 138 patients had radial-femoral artery pressure gradient after cardiopulmonary bypass (Group P) (Figure 1). Their hemodynamic data and other demographic data were analyzed.

The second box indicated that 11 patients were excluded because of a prior radial-femoral artery pressure gradient and the rest of patients did not have radial-femoral artery pressure gradient before the surgery. The third box indicated that 401 patients without pressure gradient before surgery were included in our research. The last left box suggested that 263 patients did not have radial-femoral artery pressure gradient after CPB. The last right box suggested that 138 patients had radial-femoral artery pressure gradient after cardiopulmonary bypass. 

The genders, ages, heights, weights, preoperative cardiac function, history of hypertension or diabetics, echo report about LVEDd (left ventricle end diastolic diameter), EF (ejection value), pulmonary hypertension, surgical type, clamping time, total bypass time, operation duration, vasoconstriction usage and lowest core temperature during CPB were recorded and compared between the two groups. Systolic, diastolic, and mean artery pressure in radial and femoral arteries were also recorded at baseline, during discontinuation of CPB, at the end of surgery and 1^st^ postoperative day. Pressure gradient was defined as femoral artery pressure minus radial artery pressure. Other hemodynamic data, such as systemic vascular resistance index (SVRI), pulmonary vascular resistance index (PVRI), cardiac index (CI), heart rate (HR), central venous pressure (CVP), pulmonary artery pressure (PAP), and pulmonary capillary wedge pressure (PCWP) were also monitored, calculated, and compared.

Every patient received intravenous and inhalational anesthesia. Patients were induced with midazolam (0.1mg/kg), etomidate (0.2-0.3mg/kg), vecuronium (0.15mg/kg), and fentanyl (3-5 μg/kg), and maintained with isoflurane (1-2MAC), atracurium (0.5mg/kg/h) and intermittent fentanyl (total 30-40μg/kg). Every patient received invasive artery pressure, ECG, SpO_2_ monitoring. Swan-Ganz catheter was also placed to monitor PVRI, CI, HR,CVP, PAP, and PCWP. Artery blood pressure was about baseline to 20% lower and heart rate was 60-90bpm during the surgery. The target mean artery pressure was set to 60 mmHg and the target heart rate was 90 bpm during the weaning of CPB. Fluid therapy and adrenaline were administered according to hemadynamic data such as MAP, HR, CO, CVP, PCWP, SVRI and so on. During cardiopulmonary bypass, phenylephrine was used if MAP was less than 50 mmHg in condition of adequate flow rate. All patients were regulated to normal homeostasis such as acid–base balance, electrolytes, PaO_2_ and PaCO_2_ during the entire surgical period.

### Statistic analysis

All the data were presented as means ± SD. Statistical analysis was completed by SPSS 13.0 (SPSS Inc, USA). The qualitative data were compared with Pearson chi-square analysis or Fisher’s exact test and the quantitative data were compared with one way ANOVA. The hemodynamic data were compared with repeated measures variance analysis. *P*-value less than 0.05 was considered statistical significant.

## Results

With respect to the incidence of central-peripheral pressure gradient was 50% during discontinuation of CPB and permitted error was 5%, 400 patients will be necessary to reach a statistic power of 95%. We chose totally 401 cardiac patients since those patients received operations in a certain period.

263 patients without femoral and radial artery pressure gradient were defined as group N. 138 patients with femoral and radial artery pressure gradient were defined as group P. Univariate analysis revealed that there was no significance in the gender, age, height, weight, NYHA function, history of hypertension, history of diabetics, LVEDd, EF value, pulmonary hypertension, surgical types, clamping time, total bypass time, operation time and lowest core temperature in the two groups. However, Phenylephrine usage during CPB was 1.7±1.1 mg in group N and 2.9±1.2 mg in group P(P<0.001). Total adrenaline usage was 229.2±116.9µg in group N and 400.6±145.1µg in group P (P<0.001) (Table 1 and Table 2).

There was no difference in radial and femoral SBP, DBP, MAP after anesthesia induction between the two groups, however, SBP, DBP and MAP in radial artery were significantly lower in group P than group N immediate after discontinuation of CPB, at the end of surgery and at the 1^st^ postoperative day (Table 3 and Table 4).

A minor radial-femoral pressure gradient before CPB was found in all the patients without difference between the two groups. However, significant artery pressure gradient emerged immediately after discontinuation of CPB and at the end of surgery in Group P. Artery pressure gradient almost recovered at the 1^st^ postoperative day (Table 5).

Repeated measurement variance analysis revealed higher SVRI (P<0.001) and higher PVRI (P<0.001) immediately after discontinuation of CPB to the first postoperative day. CI was lower in group P right after discontinuation of CPB, at the end of surgery and the first postoperative day compared with group N (P<0.001). CVP was higher in group P than that in group N at baseline and the first postoperative day. There was no difference in HR, PCWP or PAP between the two groups at any time point (Table 6).

**Table 1:** Demographic data of the patients.

Parameters	Group N (n=263)	Group P (n=138)	P value
Gender (male/female)	115/148	57/81	0.642
Age(years)	54.1±13.5	56.3±12.8	0.107
Height(cm)	161.1±7.3	162.1±7.7	0.216
Weight(kg)	58.3±7.4	56.8±7.0	0.067
NYHA(Ⅱ/Ⅲ/Ⅳ)	102/134/27	51/75/12	0.775
History of hypertension(yes/no)	32/231	18/120	0.801
History of diabetics (yes/no)	42/221	19/119	0.560
LVEDd (mm)	51.9±11.0	52.3±11.1	0.788
EF value (%)	58.2±7.1	57.1±7.8	0.176
PH(yes/no)	43/220	29/109	0.248

Group N: Patients without femoral-radial artery pressure gradient after cardiopulmonary bypass;

Group P: Patients with femoral-radial artery pressure gradient after cardiopulmonary bypass; NYHA: New York heart association; LVEDd: left ventricle end diastolic diameter; EF: eject fraction; PH: pulmonary hypertension

**Table 2:** Surgical and CPB data.

Parameters	Group N (n=263)	Group P (n=138)	P value
Surgical type(valve/CABG/congenital/aorta)	186/42/29/6	88/29/17/4	0.530
Clamping time(min)	68.9±20.6	70.2±23.1	0.594
Total bypass time(min)	101.4±27.1	104.1±32.8	0.387
Operation duration(min)	297.4±53.0	300.3±64.5	0.640
Phenylephrine usage during CPB(mg)	1.7±1.1	2.9±1.2	<0.001
Total adrenaline usage during the surgery (μg)	229.2±116.9	400.6±145.1	<0.001
Lowest core temperature during CPB	30.6±2.0	30.5±2.3	0.692

Group N: Patients without femoral-radial artery pressure gradient after cardiopulmonary bypass;

Group P: Patients with femoral-radial artery pressure gradient after cardiopulmonary bypass; CABG: coronary artery bridge grafting; CPB: cardiopulmonary bypass

**Table 3:** Radial artery pressure in the two groups.

	Group	After induction	Discontinuation of CPB	The end of surgery	1 postoperative day
SBP(mmHg)	N	118±15	107±7	107±7	112±8
	P	118±14	93±10##	97±9##	108±12##
DBP(mmHg)	N	56±12	50±6	51±6	56±5
	P	56±12	55±6##	54±5##	59±5##
MAP(mmHg)	N	79±10	73±6	77±5	79±5
	P	78±11	72±6#	73±6##	77±6##

#P<0.05

##P<0.01 Compared with group N

**Table 4:** Femoral artery pressure in the two groups.

	Group	After induction	Discontinuation of CPB	The end of surgery	1 postoperative day
SBP(mmHg)	N	115±14	106±7	106±9	106±10
	P	114±13	107±7	107±7	107±8
DBP(mmHg)	N	59±11	55±5	58±5	56±6
	P	58±11	54±9	59±5	55±8
MAP(mmHg)	N	79±10	76±6	77±5	76±7
	P	79±9	76±7	78±5	76±6

**Table 5:** Femoral-artery pressure gradient in the two groups.

	Group	After induction	Discontinuation of CPB	The end of surgery	1 postoperative day
SBP gradient (mmHg)	N	-3±3	0±1	-1±9	-6±4
	P	-4±3	14±9##	10±4##	0±11##
DBP gradient (mmHg)	N	3±3	5±2	7±5	0±8
	P	3±3	-1±9##	4±5##	0±8
MAP gradient (mmHg)	N	1±2	3±1	1±4	-2±5
	P	1±2	4±6	6±4##	0±6##

##P<0.01 Compared with group N

**Table 6:** Hemodynamic data in the two groups.

	Group	After induction	Discontinuation of CPB	The end of surgery	1 postoperative day
SVRI(dynes. s/cm5.m2)	N	2908.1±531.1	1965.2±362.2	2019.0±350.6	1995.4±373.9
	P	2889.6±512.0	2375.2±445.1##	2483.8±410.2##	2524.3±542.9##
PVRI(dynes. s/cm5.m2)	N	233.4±65.6	219.7±77.1	219.7±77.1	218.1±77.5
	P	252.1±88.8#	265.3±74.3##	255.9±91.8##	292.6±115.1##
CI(L/min/m^2^)	N	2.1±0.3	2.8±0.5	2.8±0.5	2.8±0.5
	P	2.0±0.3	2.3±0.4##	2.3±0.4##	2.2±0.4##
CVP(mmHg)	N	4.7±1.8	8.3±1.2	8.2±1.2	7.5±1.5
	P	5.2±2.2#	8.4±1.4	8.4±1.3	8.3±1.2#
HR(bpm)	N	88.4±12.3	92.6±4.8	92.1±5.2	92.7±4.8
	P	87.8±12.0	92.8±5.0	92.6±4.8	93.4±5.8
PCWP(mmHg)	N	7.5±2.3	8.3±2.0	8.4±2.1	8.3±2.0
	P	7.4±2.1	8.4±2.1	8.7±2.1#	8.5±2.1
PAP(mmHg)	N	13.5±3.1	15.8±2.8	15.8±2.1	15.7±2.8
	P	13.9±3.4	16.0±3.0	15.9±3.0	15.9±2.9

#P<0.05,

##P<0.01 Compared with group N

## Discussion

In our study, we demonstrated that radial-femoral pressure gradient, especially at the discontinuation of CPB was associated with constriction agents, cardiac index, SVRI, PVRI. There have been a couple of clinical studies investigated artery pressure gradient after cardiopulmonary bypass prospectively. Some of them found that the pressure gradient was associated with bypass time, body temperature, and catecholamine levels [4–7], but others not [2,3]. It is difficult to estimate whether a patient will have pressure gradient or not. Our study is appropriate to figure out the relative risk factors associated with significant pressure gradient. In our study, we picked out patients with pressure gradient. The pressure gradient of some patient was significant, which made it applicable to investigate the risk factors involved in pressure gradient.

Artery pressure wave is based on the cardiac output and artery elasticity. Previous studies found that the artery pressure gradient was due to the artery elasticity [8,9]. In our study, we cannot make any decision about the artery elasticity as there was no specific method to measure the systemic vascular elasticity during cardiac surgery. And also the possibility of the systemic vascular elasticity changing from anesthesia to discontinuation of CPB is small.

Increased SVRI and PVRI contributed to significant pressure gradient. It was consistent with those studies in which vasodilating therapy was beneficial to artery pressure gradient [10,11]. Normally, SVRI is seldom determined by arteries located between aorta to radial arteries. Conversely, SBP will increase a little bit due to peripheral pressure reflex wave. Ultrasonagraphy had disclosed decreased radial artery diameter in patients with significant artery pressure gradient [7]. Although we did not measure the radial artery diameter with ultrasonagraphy, we can hypothesize that peripheral artery diameter will become decreased in lower cardiac function patient since literature reported that flow-mediated radial artery dilatation was suppressed in heart failure models [12,13] and the possible mechanism was associated with vascular endothelium dysfunction [14]. However, some investigators found that post-CPB artery pressure gradient was not affected by vascular resistance changes induced by vasoconstriction or vasodilation [3,15]. And even Urzua J reported that vasodilation was the main cause of radial-femoral artery pressure gradient after cardiac surgery [16]. From our results, we can explain that systemic vascular resistance is not a correlated factor of pressure gradient but radial artery pressure gradient after bypass is a multifactorial event.

Literatures [17,18] and our study demonstrated that PVRI would be increased after CPB, however, in our study, PVRI was also associated with pressure gradient. Both the literature and our results suggested a pulmonary vascular relaxing strategy were very important in these patients.

We regard femoral artery pressure as the central artery pressure because femoral artery pressure is more similar to the central artery pressure than radial artery pressure. And also previous researches have successfully investigated femoral artery to radial artery pressure gradient as central-peripheral pressure gradient after cardiopulmonary surgeries [15,16,19,20].

Our reviewed patients had both femoral artery and radial artery pressure monitoring, which indicated that these patients may have some prior problems such as weaker radial pulsation, poor peripheral vascular perfusion, or difficult radial artery catheter placement. So these patients might be easier to present artery pressure gradient after cardiopulmonary bypass. That’s probably why there were many risk factors in our results and other prospective or retrospective studies did not.

In conclusion, when patients have artery pressure gradient after cardiac surgery, detecting the exact artery pressure is most important. Real artery pressure makes it possible to calculate the exact hemodynamic parameter. Inotropic agents, avoiding vasoconstriction might ameliorate artery pressure gradient.
